# Retention Time and Fragmentation Predictors Increase Confidence in Identification of Common Variant Peptides

**DOI:** 10.1021/acs.jproteome.3c00243

**Published:** 2023-09-01

**Authors:** Dafni Skiadopoulou, Jakub Vašíček, Ksenia Kuznetsova, David Bouyssié, Lukas Käll, Marc Vaudel

**Affiliations:** † Mohn Center for Diabetes Precision Medicine, Department of Clinical Science, University of Bergen, NO-5020 Bergen, Norway; ‡ Computational Biology Unit, Department of Informatics, University of Bergen, NO-5020 Bergen, Norway; § Institut de Pharmacologie et de Biologie Structurale (IPBS), Université de Toulouse, CNRS, Université Toulouse III—Paul Sabatier (UT3), 31000 Toulouse, France; ∥ Science for Life Laboratory, School of Engineering Sciences in Chemistry, Biotechnology and Health, KTH Royal Institute of Technology, SE-100 44 Stockholm, Sweden; ⊥ Department of Genetics and Bioinformatics, Health Data and Digitalization, Norwegian Institute of Public Health, N-0213 Oslo, Norway

**Keywords:** proteogenomics, single amino acid variation, peptide identification, peptide feature predictors

## Abstract

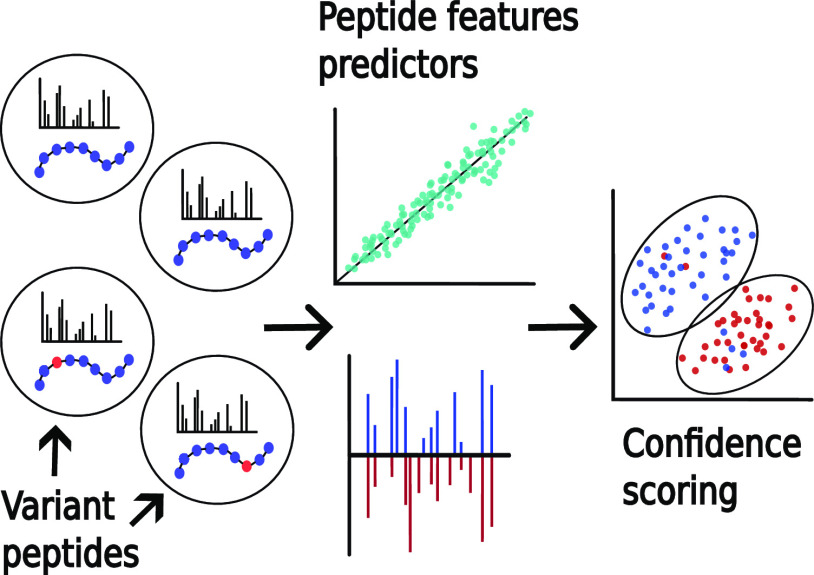

Precision medicine focuses on adapting care to the individual profile of patients, for example, accounting for their unique genetic makeup. Being able to account for the effect of genetic variation on the proteome holds great promise toward this goal. However, identifying the protein products of genetic variation using mass spectrometry has proven very challenging. Here we show that the identification of variant peptides can be improved by the integration of retention time and fragmentation predictors into a unified proteogenomic pipeline. By combining these intrinsic peptide characteristics using the search-engine post-processor Percolator, we demonstrate improved discrimination power between correct and incorrect peptide-spectrum matches. Our results demonstrate that the drop in performance that is induced when expanding a protein sequence database can be compensated, hence enabling efficient identification of genetic variation products in proteomics data. We anticipate that this enhancement of proteogenomic pipelines can provide a more refined picture of the unique proteome of patients and thereby contribute to improving patient care.

## Introduction

Genomic variation can affect proteins, their expression, structure,^1^ degradation rates, or even completely prevent their production.^2^ Consequently, cellular functions can be altered, possibly participating in the development of diseases.^3^ Therefore, monitoring the proteomic profiles of patients is seen as a promising technique for the development of precision medicine approaches.^4^ However, in mass spectrometry (MS)-based proteomics, spectra are usually matched to a one-database-fits-all set of protein sequences. Projecting all data onto a database that does not capture the diversity of proteomic samples can yield false positive identifications,^5^ but more importantly, it creates a bias toward populations of study participants based on their genetic similarity with the reference database.

The personalization of proteomic searches using genomic information is an active field of research in proteogenomics.^6^ Typically, proteomic MS data are matched against a database of sequences capturing the products of genomic sequence variation. These databases can be constructed based on genomic or transcriptomic sequencing data or, when no genomic data are available, using variants from knowledge bases like Ensembl.^7^ However, expanding protein sequence databases using sequence variation poses major challenges to the current bioinformatic methods for protein identification: (i) the search space of possible peptides used to match spectra is enlarged, yielding higher processing time and increasing the likelihood of matching a false positive at a given score^8^ and (ii) variant peptides containing the product of an amino acid substitution are highly similar to canonical or modified peptides and thus difficult to confidently identify.^9−11^ These issues, combined with the low sequence coverage of proteomics, make the detection of the products of genetic variation a challenging task, with recent publications showing low identification rates of variant peptides compared to what was expected after analysis at the DNA and RNA level.^12,13^ And when variant peptides are matched to spectra, the evaluation of results remains challenging, often requiring costly experimental validation.^12^

The confidence in peptide identification is evaluated by search engines through the matching of the measured spectra with expected fragment ions and returned as a score. The score is translated as a statistical metric, for example, a false discovery rate (FDR), *e*-value, or posterior error probability, after comparison with the estimated null distribution of scores. The reference method for the estimation of the null distribution of scores is the target-decoy strategy, where incorrect sequences, the *decoy* sequences, for example, randomized, shuffled, or reversed, are inserted in the database and compete equally with the sequences of interest, the *target* sequences.^14^ However, these scores rely on limited information on the peptides, typically only predicted fragment masses, and usually only consider the most intense peaks in the measured spectra. Bioinformatic approaches were therefore developed that allow re-scoring the matches based on more peptide features and implemented in bioinformatic tools like Percolator,^15^ Scavager,^16^ and AlphaPept.^17^ Notably, the inclusion of predicted retention time^18,19^ and predicted intensities of fragment ions^20−23^ have been demonstrated to increase spectrum identification rates, for example, with application in immunopeptidomics.^24^

In this work, we investigate how the inclusion of common germline variations affects the performance of proteomic searches. We demonstrate how variant and canonical peptides distribute in the predicted retention time and fragmentation feature space and how these can be used to increase the share of confidently identified variant peptides. Together, our results show that with careful curation of the protein sequence database and using the available tools for post-processing MS data, we can gain better coverage of the variation of the proteome.

## Experimental Section

### Data Samples

The processed samples were published by Wang et al.^12^ and downloaded from the PRIDE repository^25^ with the identifier PXD010154. From this dataset, the chosen subset of samples consists of 106 MS raw files of healthy tonsil tissues acquired from 3 different experiments with identifiers P010747, P010694, and P013107. Briefly, the proteins were digested with trypsin and analyzed by tandem MS coupled with liquid chromatography (LC–MS/MS) using a Q Exactive Plus mass spectrometer (Thermo Fisher Scientific, Bremen, Germany) coupled to a nanoflow LC system (NanoLC-Ultra 1D+, Eksigent, USA) using a 110 min gradient, yielding 5,085,477 MS/MS spectra (Exp. P010747: 1,834,613 MS/MS spectra, Exp. P010694: 1,695,460 MS/MS spectra, Exp. P013107: 1,555,404 MS/MS spectra). MS1 scans were acquired at a resolution of 70,000, and MS2 scans were acquired for up to 20 precursors after HCD fragmentation. For more details on the data generation, please refer to the original publication by Wang et al.^12^ A quality control of the raw data was performed using the software tool viQC,^26^ and the results for each used raw file can be found at the GitHub repository^a^.

### Protein Databases

The search was done against four different protein databases, three that included the canonical human proteome and/or protein isoforms and one that also included genetic variation products. The three canonical databases were (i) the *homo sapiens* complement of the UniProtKB^27^ database downloaded on September 20, 2022 (20,398 distinct protein sequences), (ii) the *homo sapiens* complement of the UniProtKB database including protein isoforms downloaded on January 9, 2023 (42,397 distinct protein sequences), and (iii) the canonical database of protein isoforms of homo sapiens taken from Ensembl v.104^28^ (92,558 distinct sequences).

The extended database included the protein products of genetic variants, appended with the canonical database of protein isoforms taken from Ensembl v.104 (248,518 distinct sequences). We included variants with a minor allele frequency >1%, taken from Ensembl v.104, and six-frame translations of variant cDNA were obtained using the Python tool py-pgatk.^7^ We then included only translations of the main open reading frame (mORF) in each transcript, as annotated per Ensembl v.104. Translations of cDNA without an annotated mORF were not included in the database. Decoy sequences were generated using the algorithm DecoyPYrat,^29^ implemented by py-pgatk.

All databases were supplemented with sample contaminants from the common Repository of Adventitious Proteins (cRAP, thegpm.org/crap). In order to compare the tryptic peptides contained in the extended database with those in UniProtKB, both databases were digested in silico following the cleavage pattern of trypsin with up to two missed cleavage sites, retaining peptides of length between 8 and 40 residues. The two lists of peptide sequences were then merged, and each peptide was assigned a list of proteins between which the peptide is shared. Peptides shared between the extended database and UniProtKB were labeled.

### Proteomic Search

The RAW files were converted to mzML files using ThermoRawFileParser version 1.3.4.^30^ The mzML files were searched using the X!Tandem search engine^31^ operated through the SearchGUI interface version 4.0.41.^32^ Search settings were (1) specific cleavage by trypsin with a maximum number of 2 missed cleavages; (2) carbamidomethylation of C as fixed and oxidation of M, deamidation of N and Q, and acetylation of protein N-terminus as variable modifications; (3) peptide maximum length of 40 amino acids; and (4) precursor and fragment ion tolerance of 10 ppm. The refinement step of X!Tandem was disabled. PeptideShaker version 2.2.25^33^ was used to process the output of X!Tandem and generate standardized PSM exports.

### Peptide Feature Predictors and Confidence Scoring

Percolator^15^ version 3.5 was used for the statistical evaluation of the resulting peptide-to-spectrum matches (PSMs). For each PSM, a set of features commonly used for Percolator^34^ was generated using PeptideShaker, referred to as the *standard* set of features, and described in Supporting Information, Table 1. This standard set of features was extended with novel features capturing the agreement between PSMs and predicted retention times and fragmentation, resulting in a new set of features referred to as the *extended* set of features and described in Supporting Information, Table 2.

DeepLC version 1.1.2^19^ was used to compute predictions of the retention time of each theoretic peptide of each PSM. To tackle the problem of the large range of possible elution times of a peptide, the peak of the elution (i.e., RT apex) was used for each PSM instead of the time of MS2 acquisition. For each spectrum, the RT apex was calculated with the software tool Proline,^35^ and in case the apex was not found, the measured retention time as available in the spectrum files was used instead. The retention times of the confident (*q*-value ≤0.01) PSMs according to Percolator using the standard features were used to calibrate the predictions of DeepLC. These were then compared with the retention time apex reported for the matched spectrum and three different metrics (absolute distance, square distance, and logarithmic distance) were calculated and used as PSM features. An additional feature was the absolute distance between the measured RT and the apex. The retention time error used in the figures of the Results section correspond to the residuals of a linear regression model computed on the RT apex and predicted retention times of the confident target hits from Percolator run using the standard set of features.

The peptide fragmentation predictions were obtained from MS^2^PIP version 3.6.3^21^ using the HCDch2 pretrained model and were compared against the peaks of the experimental spectrum after matching the predicted and observed fragment peaks using a 10 ppm threshold and the normalization of the intensities of the peaks. The features used to evaluate the concordance between experimental and predicted spectra were (1) the percentage of predicted peaks matched with an observed one, (2) the logarithmic distance, (3) the cosine and angular similarity, and (4) the cross entropy between the spectra. These features were calculated when taking into account the predicted b and y ions separately and also with all ions combined. In addition, the number of consecutive amino acids matched from the N and C termini were computed for the b and y ions, respectively.

### Code Availability

All steps of the proteogenomic pipeline described above are implemented in a Snakemake^36^ workflow (version 6.8.0). The post-processing of the results of Percolator and the creation of the figures were conducted using custom scripts available at the GitHub repository of the paper. A list with the required software packages together with further documentation and links to supplementary data are also included in that GitHub repository. The used databases and other supplementary data are available in Zenodo (doi:10.5281/zenodo.8214353)

## Results

To investigate the influence of including germline variation on the performance of proteomic search engines, four different protein sequence databases were used, the standard UniProtDB, UniProt with isoforms, Ensembl with isoforms, and Ensembl with isoforms extended with common amino acid substitutions. Three samples of healthy tonsil tissue by Wang et al.^12^ were searched against these four databases using X!Tandem.^31^ The identification results from X!Tandem were then post-processed by Percolator^15^ using a set of features proposed in the literature.^34^ See the Experimental Section for details.

### Including Germline Variation Does Not Impair the Identification Rate

**Figure 1:**
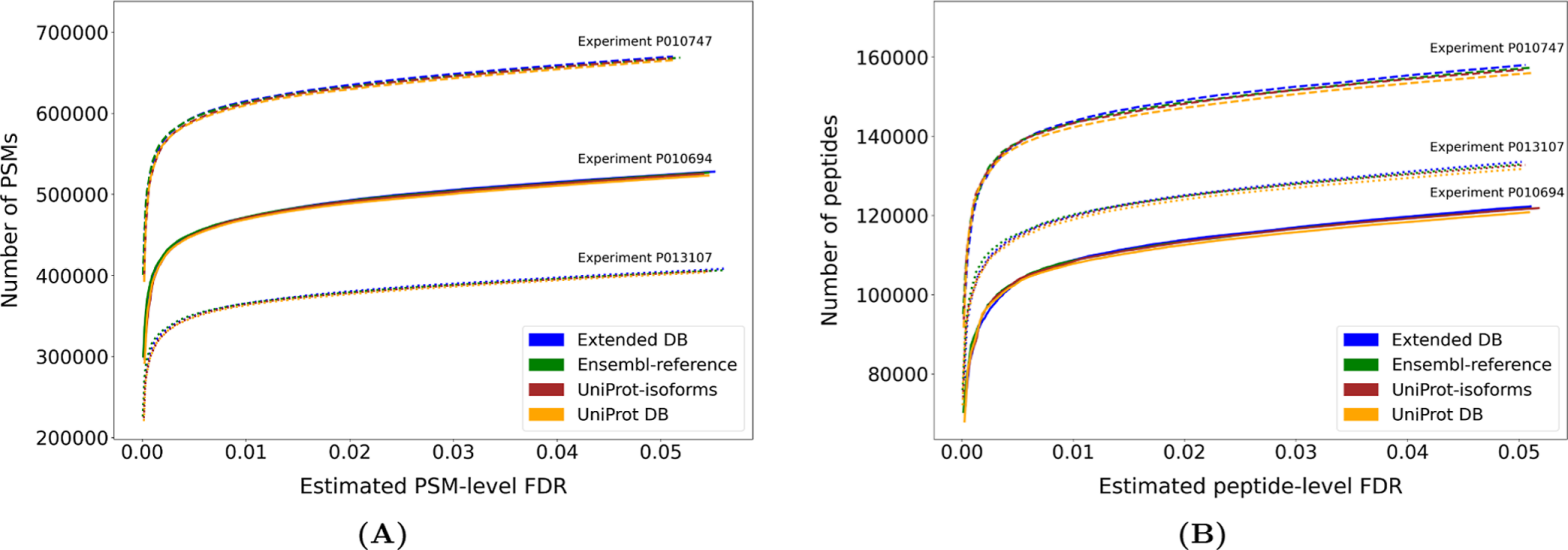
Comparison of the performance when matching spectra to reference databases and extended databases. We searched three different sets of spectra against four different human protein sequence databases. The number of hits obtained at a given FDR threshold is displayed for (A) PSMs and (B) peptide sequences.

When germline variation and isoforms are included in the database, there is a substantial increase in the number of sequences that the search engine has to match each spectrum with. In such a case, one would expect a decrease in search performance. However, identification rates at a given FDR were nearly identical for the different databases for all three tonsil samples from Wang et al.^12^ for both PSMs and peptides (Figure 1A,B, respectively). In our hands, the different tonsil experiments yielded different numbers of PSMs and peptides (Figure 1). While we could not explain the source of this difference in yield between experiments, the performance was consistent for all databases in all experiments.

The similar or even better performance displayed by the variant-aware Ensembl database indicates that the increase in the number of protein sequences does not create a massive increase in the number of peptides that can match a spectrum. Indeed, after an in-silico digestion of the extended database, 76.15% of the tryptic peptides were canonical sequences included in UniProt DB, and only 23.85% were newly introduced peptide sequences. This is also reflected by very similar score distributions for the searches against the canonical UniProt database and the extended Ensembl one (Supporting Information Figure S4). The high level of similarity between isoforms and variant proteins might explain that a high number of sequences does not result in a much enlarged search space, in contrast to, for example, including three-frame translations of untranslated regions (UTRs) or non-coding sections of the genome. Together, these results demonstrate that extending proteomic sequence databases using common germline variation does not compromise identification rates while enabling a broader coverage of populations.

### Fragmentation and Retention Time Prediction Allows Discriminating Random Matches

Introducing variant sequences, however, increases the risk of one peptide being difficult to distinguish or even identical to another, possibly from a different protein. The mass difference of an amino acid substitution might even be indistinguishable from a chemical or post-translational modification, yielding equal search scores for the two versions of a peptide that can be encoded by this variant.^9^ This further increases the need for post-search validation of the identifications that can tease apart highly similar peptides. Such tools take advantage of a large number of features to evaluate the quality of PSMs. The features are based on characteristics of the peptide and the spectrum, such as the length of the peptide or the difference between the measured and the theoretic mass over charge ratio of the precursor and fragment ions. In case two peptidoforms, for example, a variant and a modified peptide, have the exact same atomic composition, they will be indistinguishable by mass. But if such peptidoforms elute at different retention times or produce fragments of different intensities, and if such differences can be tracked by state-of-the-art predictors, then these can be used to distinguish true from false hits. These two characteristics are therefore expected to be a valuable source of information to evaluate the confidence in variant peptide estimations.

**Figure 2:**
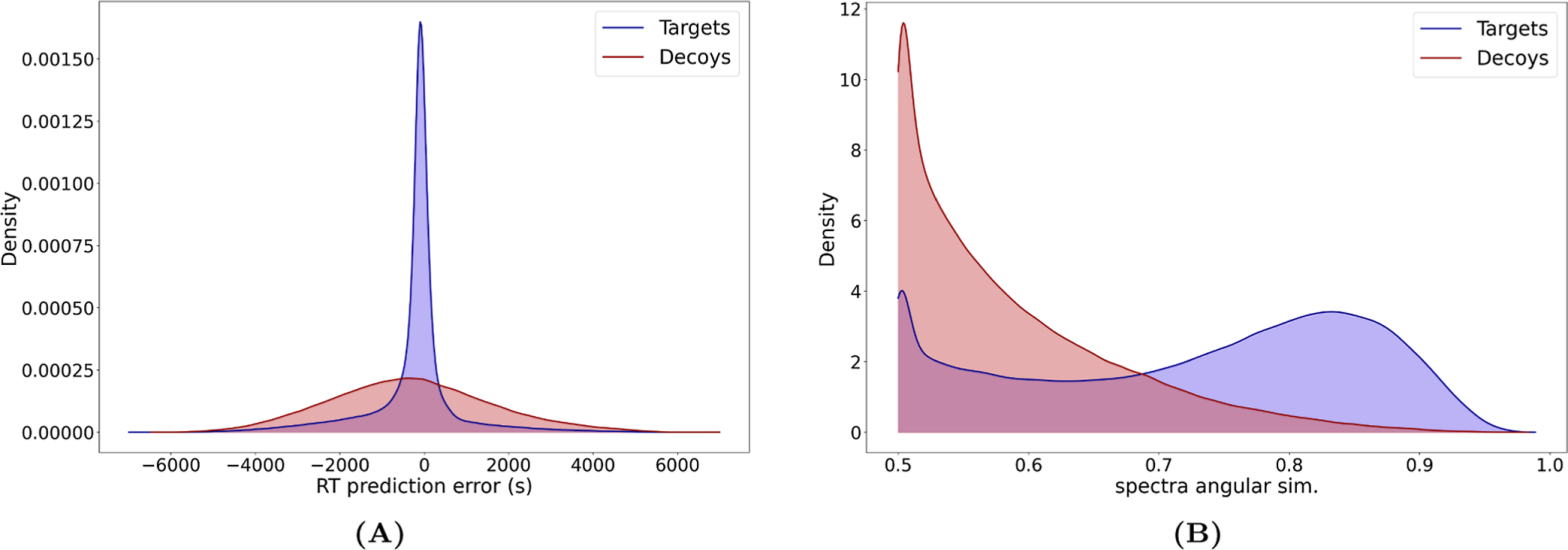
Comparison of PSM feature distributions between target and decoy sequences. Density plots that compare the distributions of the retention time prediction error and spectra angular similarity between measured and predicted values of target and decoy hits from the extended DB. PSMs pooled from the 3 used samples.

For each PSM obtained on the tonsil data by Wang et al.,^12^ we compared the measured values of retention time and fragmentation with predictions made by DeepLC^19^ and MS^2^PIP,^21^ see the Experimental Section for details. For peptide retention times, the distances between measured and predicted values were wider for decoys than for target peptides, confirming that excluding PSMs with high deviation in retention time compared to prediction would reduce the prevalence of random matches (Figure 2A and Supporting Information, Figures S5A and S6A). It is important to note that both distributions are centered close to zero, which means that there is a substantial number of decoy hits where by chance the retention times expected to be measured for the set of amino acids of these decoy sequences are very close to the measured retention times of the corresponding spectra (Supporting Information, Figure S3).

For peptide fragment intensities, target hits had a higher share of PSMs with a high similarity between the measured and predicted spectra, and most decoy hits had a very poor agreement between the measured and predicted spectra (Figure 2B and Supporting Information, Figures S5B and S6B). This can be explained by the fact that several peptides of different compositions may coelute but still fragment differently; fragmentation patterns are thus more discriminative than retention times. A random match is therefore much less likely to present a good spectrum similarity than a low retention time difference. This indicates that selecting PSMs with high similarity would enrich the dataset for high-quality matches. For many PSMs, no measured peak could be matched to predicted peaks, yielding the lowest similarity score (0.5) (117,580 PSMs from the variant-aware Ensembl database: 85,004 targets and 32,576 decoys). This can be due to a completely wrong match or to the predictor failing to predict the intensity of some peaks for the given peptide. Given the high prevalence of decoys with very low similarity scores, it can be anticipated that most PSMs with low scores will be incorrect matches, but one cannot rule out that some good matches will present low similarity scores due to the performance of the fragmentation predictor.

For both retention time and peptide fragmentation, no relevant difference was observed between the different databases (Supporting Information, Figures S5 and S6). The similarity of the distributions of the investigated features for the four sequence databases’ decoy PSMs indicates that there is no obvious bias between the databases. Also, the nearly identical distributions of target PSMs confirms that using the larger databases does not substantially increase the prevalence of hits of lower quality.

**Figure 3:**
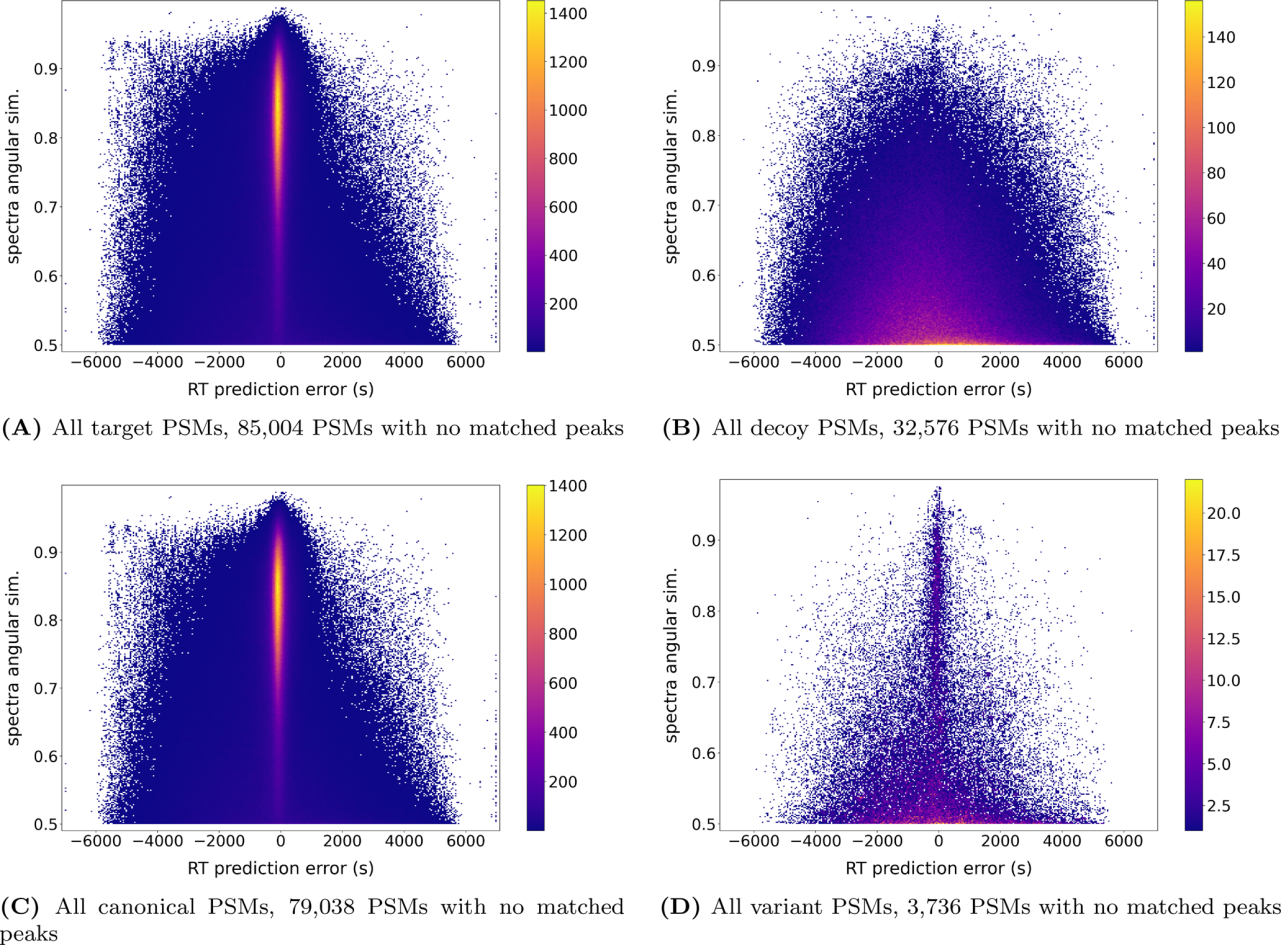
2D-density plot of PSM agreement with retention time and fragmentation predictors. The retention time vs fragmentation distance to prediction of all (A) target, (B) decoy, (C) canonical, and (D) variant PSMs obtained when searching against the extended protein sequence database. PSMs with no matched peaks are not represented, and their prevalence is listed under the plot. Pooled PSMs from the 3 used samples are presented.

When focusing on the joint distributions of both PSMs’ features, for the results obtained on the variant-aware database, as expected from Figure 2, the decoy hits distribute symmetrically around the zero retention time deviation, with most hits at the lowest spectrum similarity, with the density of hits decreasing with the similarity. The distribution of RT errors of decoy hits did not seem to depend on the angular similarity (Figure 3B). On the other hand, the target hits display a similar background of hits supplemented with a dense cloud of PSMs with a small retention time deviation and high spectrum similarity, which is likely to contain the best matches. When separating the target PSMs between those mapping to a canonical protein (97.6%) and those solely mapping to variant peptides (2.4%), one can see that for medium to low spectra similarities the distribution of variant PSMs resembles that of decoy hits, with the most dense area being close to a spectra similarity of 0.5 and spanning to a broad range of RT prediction error centered around zero (Figure 3D). However, there is also a marked cloud of PSMs with a high spectrum similarity (upper part of the plot) where the RT prediction error is very small for the majority of the hits. This demonstrates that even though the variant peptides present a higher prevalence of PSMs presenting poor agreement with the predictors than the canonical PSMs, they also have a substantial share of high-quality matches. Therefore, the agreement with predictors can help discriminate them from the random matches.

### Percolator Combined with Predictors Increases the Identification Rate of Variant Peptide Sequences

**Figure 4:**
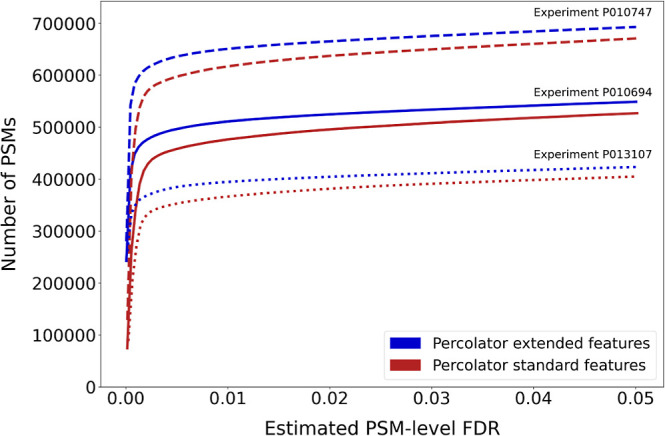
Comparison of the performance of Percolator given the standard and the extended set of features. For all three different sets of spectra that were searched against the extended protein sequences database, the number of PSMs retained at a given FDR threshold is plotted using the standard and extended sets of features for all thresholds up to 5% FDR.

**Figure 5:**
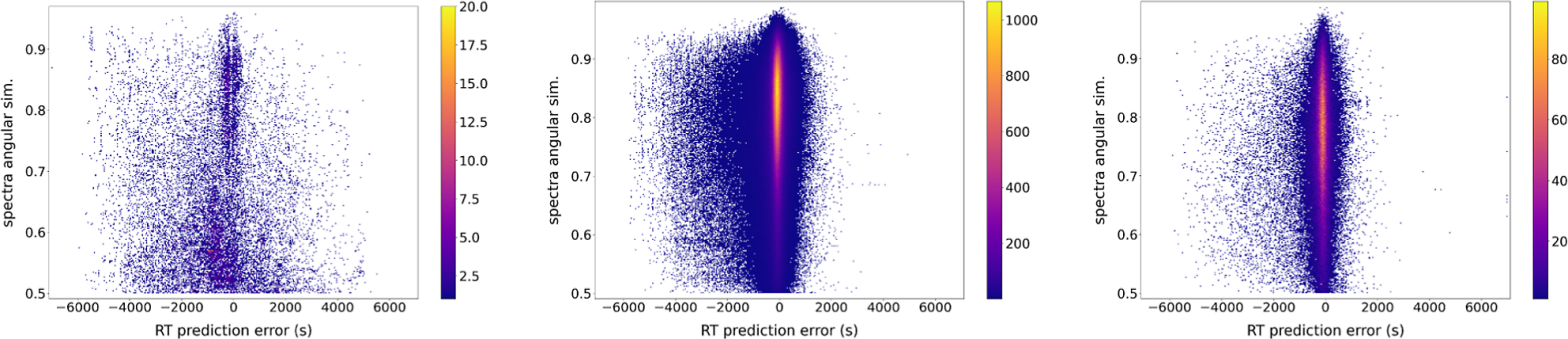
2D-density plot of PSM agreement with retention time and fragmentation predictors for confident PSMs separated based on the set of features supporting their identification. The retention time vs fragmentation distance to prediction of target PSMs retained at a 1% FDR when Percolator was provided with (A) only the standard set of features, (B) either the standard or extended set of features, and (C) only the extended set of features. PSMs pooled from the 3 used samples.

As demonstrated in ref (24), since the retention time and fragmentation pattern features capture different aspects of the quality of the match between a spectrum and a theoretical peptide, their inclusion can enhance the discriminative power of Percolator. We investigated whether this increased performance would improve the identification of the product of germline variation, which is particularly challenging due to its similarity with the reference proteome. We extended the set of features given to Percolator to capture the agreement between experimental peptide retention time and fragmentation and predicted values, making a total of 40 features compared to 18 in the standard set (full list of PSM features available in Supporting Information). For all three tonsil samples from Wang et al.,^12^ and despite performance differences in the overall yield, identification rates were consistently improved when using the extended features (Figure 4). At a global 1% FDR threshold, Percolator using the new set of features increased the prevalence of PSMs with low retention time and fragmentation deviation from the predicted values and rejected PSMs with poor retention time or fragmentation pattern matching (Figure 5).

**Figure 6:**
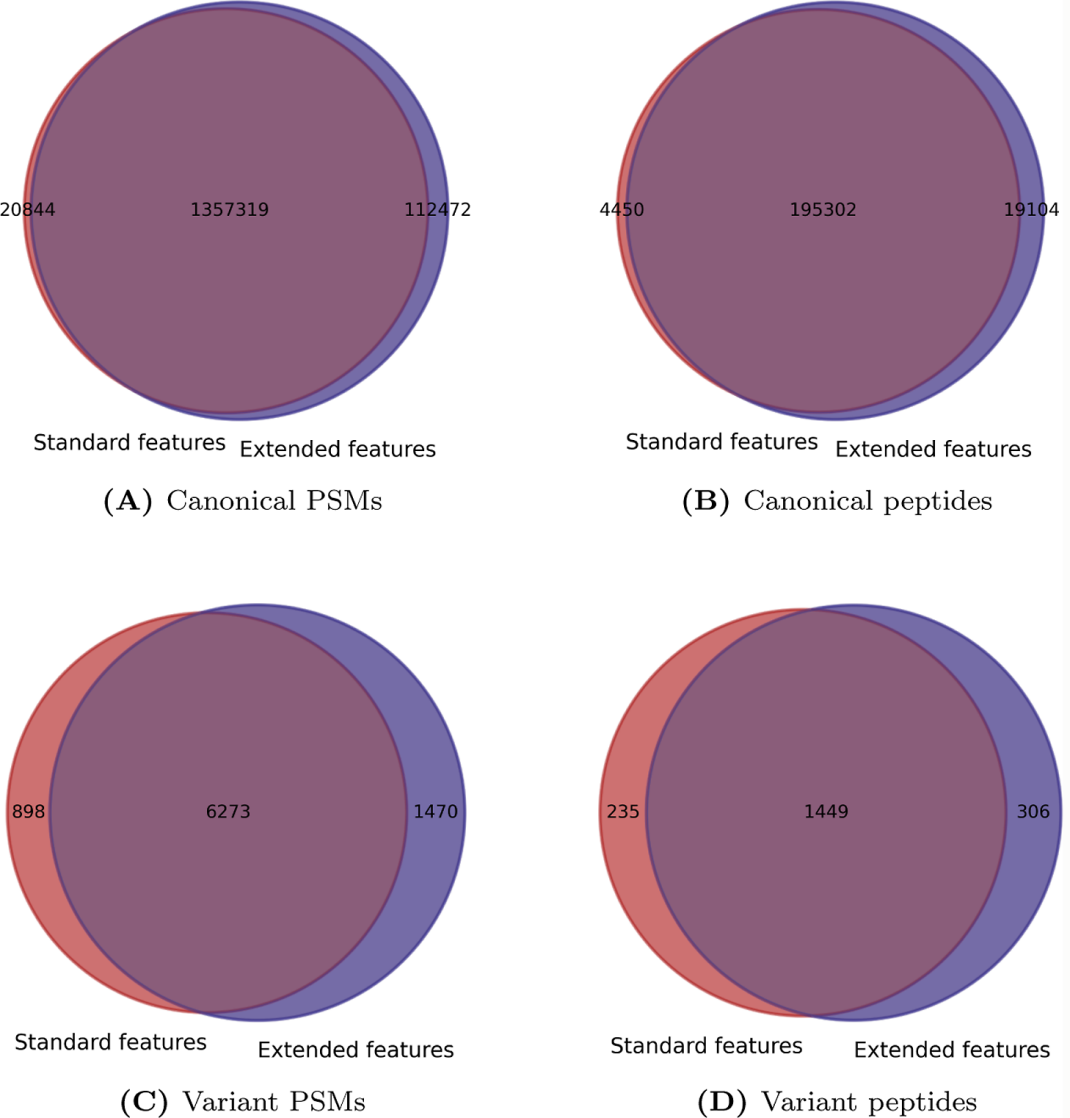
Venn diagrams of the number of PSMs and peptide sequences obtained using different sets of features. The number of PSMs and peptide sequences retained using the standard set of features only, either the standard or extended set of features, and the extended set of features only are provided for canonical and variant sequences, as listed in Table 1. PSMs and identified peptides pooled from the 3 used samples.

When summarizing the identifications from all three samples, for peptides mapping to a canonical protein sequence, 20,844 PSMs (1.5%) of the original matches were not retained using the extended features and 112,472 were newly included, representing an increase of 6.65% (Figure 6 and Table 1). When considering distinct peptide sequences, 4,450 sequences (2.2%) were not retained and 19,104 were newly included, yielding a 7.3% increase. For variant peptides, 898 PSMs (12.5%) were not retained and 1,470 PSMs were newly included, making a 8% increase. When considering distinct peptide sequences, 235 sequences (14%) were not retained and 306 were newly included, making a 4.2% increase. Thus, using the extended features increased the identification rates for all matches.

**Table 1:** Number of Matches Retained by Percolator Using Different Sets of Features^a^

	standard feature set	both feature sets	extended feature set
canonical PSMs	20,844	1,357,319	112,472
canonical peptides	4450	195,302	19,104
variant PSMs	898	6273	1470
variant peptides	235	1449	306

a The number of PSMs and peptide sequences retained using the standard set of features only, either the standard or extended set of features, and the extended set of features only are provided for canonical and variant sequences.

Even though the share of variant PSMs and peptide sequences that are gained by the extended set of features is slightly smaller for variant sequences than canonicals, there is a substantially larger percentage of variant PSMs and peptide sequences that are not retained from the standard search. Therefore, the proposed approach manages to eliminate a larger share of random hits mapping to variant sequences from the final confident identifications. Given that variant peptides can be more difficult to distinguish from others, for example, due to post-translational modification, it is expected that these will benefit best from an increased ability to assess the quality of a match. The agreement between PSMs and peptide sequences further indicates that the increase is not only due to the redundant sampling of the same sequence.

**Figure 7:**
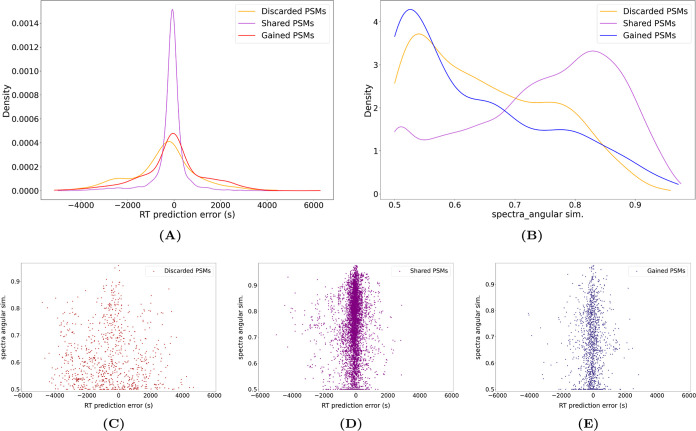
Density plots of the variant PSMs accepted by Percolator using different sets of features. (A,B) Density plots that show the PSM feature agreement with the predictors for variant PSMs, resulting from Percolator using (A) standard vs {standard + fragmentation} features or (B) standard vs {standard + retention time} features. Discarded PSMs are the ones that were only accepted by the standard features set. Shared PSMs are the ones accepted both by the standard set and (A) {standard + fragmentation} features set or (B) {standard + retention time} features set. Gained PSMs are the ones that were only accepted by (A) {standard + fragmentation} features set or (B) {standard + retention time} features set. (C–E) 2D-density plots of the retention time vs fragmentation distance to prediction of variant PSMs retained at a 1% FDR when providing Percolator with (C) only the standard set of features, (D) either the standard or extended set of features, and (E) only the extended set of features, where the extended set of features comprises {standard + retention time + fragmentation} features. PSMs pooled from the 3 used samples.

The variant PSMs that are not retained using the extended features are mainly the ones that show a large disagreement with the prediction for retention time and/or fragmentation, which makes them less reliable (Figure 7C). While, on the other hand, the PSMs that are gained (Figure 7E) together with the ones that are accepted by both sets of features (Figure 7D) display a much better agreement with the predictors: for the majority of them, the retention time of the measured spectrum is very close to the predicted one, and also intensities of the measured spectrum are highly similar to the predicted fragmentation pattern of the theoretic peptide. Extending the features therefore not only increases the identification rate for variant peptides, it also improves the agreement with predicted retention time and fragmentation. When extending the standard features with only fragmentation pattern or retention time information, the gain in retention time or fragmentation agreement is minimal, as shown in Figure 7A,B, respectively. This further supports the need to extend the features both with retention time and fragmentation features, as these two have a complementary contribution to Percolator’s rescoring procedure. Therefore, the gained PSMs are more reliable when both peptide characteristics are used, rather than either of them separately.

## Conclusions and Discussion

This work focuses on the search for the product of common genetic variation in proteomic data. For this purpose, we evaluated the combination of retention time and fragmentation predictors (Supporting Information, Figure S1). We tested the performance of this approach on a dataset of healthy tonsil tissue samples available from Wang et al.^12^ Testing on a peer-reviewed reference public dataset provides the advantage of generating independent results that should be better generalizable to other proteogenomic datasets. The search was performed against an Ensembl-based protein database, enriched with the products of common genetic variants and sample contaminants. In order to improve the identification rate for canonical and especially variant sequences, the retention time and fragmentation pattern of the peptides were used for the computation of additional features for Percolator. The results presented in this paper show that there is indeed a significant influence of these two characteristics on the outcomes of our analysis. By taking them into account, Percolator is able to retrieve a set of accepted PSMs with a greater prevalence of high-quality matches, leading to an increased number of identified peptide sequences.

Combining different features for peptide scoring and evaluation to improve the filtering of false positives has been used since the early days of mass spectrometry-based proteomics,^22,37−39^ and modern prediction tools have enabled the routine usage of retention time and fragmentation predictors for PSM rescoring. Notably, the Prosit rescoring method^40^ and MS^2^Rescore^24^ demonstrated impressive performance improvements for the identification of immunopeptides. Given the intrinsic difficulty in identifying variant peptides, we here evaluated the value of adding features capturing agreement with retention time or/and fragmentation predictors to proteogenomic pipelines. Our results strongly encourage the usage of such state-of-the-art PSM rescoring tools in proteogenomics searches, as this allows the identification of more unique peptide sequences while also increasing the quality of the matches between spectra and peptides, leading to a broader coverage of the proteome. For this, proteogenomic pipelines can be extended with tools featuring the built-in support of such predictors like Prosit^40^ or MS^2^Rescore.^24^ Further gain is expected from the tuning of the features of such tools for proteogenomic applications, notably to distinguish variant peptides from similar and possibly modified reference peptides, which was beyond the scope of our study.

The performance of peptide identification search engines is strongly affected by the protein sequence database used. In this work, the focus was on products of common germline sequence variations which increase the database size but do not yield a search space explosion compared to rare or somatic mutations. If rare (MAF ≤ 1%) or somatic variants were also included, then for most of the proteins there would be orders of magnitude more unique sequences that would need to be included in the extended database. Similarly, if the products of UTRs or non-coding variants were included, then the massive size of the resulting database would pose several challenges, and the prevalence of false positive hits would increase significantly. In these cases, the improvement of Percolator’s evaluation with the additional features of retention time and fragmentation pattern is likely to also have a positive influence on the performance of a proteogenomics pipeline. Another factor impacting the identification of common germline variants in comparison to somatic and non-coding peptides is the fact that, by nature, these are very similar to reference peptides and unlikely to alter the function of proteins or be pathogenic. Common variant peptides are thus more likely to be mistaken with another peptide, and such errors are less likely to be monitored by error rates using random peptides to model the null distribution of scores.

Our study focused solely on the identification of PSMs and peptide sequences. Accounting for common germline variation remains to be integrated with PTM detection and localization methods to enable the identification of peptides. Similarly, new methods and tools need to be developed to consolidate variant-aware peptide information at the gene or protein level. But overall, our results support that current proteomic pipelines have the potential to account for products of germline genetic variants. Routinely including genetic variation in proteomic analyses holds the promise to increase their value in medical and population studies, and especially in precision medicine approaches. It also provides a simple alternative to projecting all data onto an arbitrary reference genome, hence enabling a better and fairer coverage of populations.

## Supporting Information Available

List of PSM describing features included in the standard features set given to Percolator as input; list of PSM describing features included in the extended features set given to Percolator as input; schematic representation of the proposed proteogenomics pipeline; prediction errors for confident target PSMs; comparison of predicted retention time and RT apex for decoy and confident target hits; *Q*–*Q* plot of search engine score distributions between extended and canonical databases; *Q*–*Q* plots of PSM features distributions between extended DB and UniProt DB; and violin plots of PSM features distributions between extended and canonical databases (PDF)
Standard and extended sets of features representing PSMs (XLSX)

## Footnotes

a https://github.com/ProGenNo/VariantPeptideIdentification.
