# Supplementary material for: Retention Time and Fragmentation Predictors Increase Confidence in Identification of Common Variant Peptides
Source: J Proteome Res. 2023 Sep 1;22(10):3190–9. doi: 10.1021/acs.jproteome.3c00243 (PMC10563157; doi:10.1021/acs.jproteome.3c00243)
Supplement: Supplementary file 1 — pr3c00243_si_001.pdf [file pr3c00243_si_001.pdf]

Supplementary material: Retention time and fragmentation predictors increase confidence in identification of common variant peptides

Dafni Skiadopoulou[1,2] Jakub Vašíček[1,2] Ksenia Kuznetsova[1,2]
David Bouyssié[3] Lukas Käll[4,†] Marc Vaudel[1,2,5,†,*]

[1]Mohn Center for Diabetes Precision Medicine, Department of Clinical Science, University of Bergen, NO-5020 Bergen, Norway
[2]Computational Biology Unit, Department of Informatics, University of Bergen, NO-5020 Bergen, Norway
[3]Institut de Pharmacologie et de Biologie Structurale (IPBS), Université de Toulouse, CNRS, Université Toulouse III - Paul Sabatier (UT3), 31000 Toulouse, France [4]Science for Life Laboratory, School of Engineering Sciences in Chemistry, Biotechnology and Health, KTH Royal Institute of Technology, SE-100 44 Stockholm, Sweden
[5]Department of Genetics and Bioinformatics, Health Data and Digitalization, Norwegian Institute of Public Health, N-0213 Oslo, Norway,
[†]These authors jointly supervised the work
[*]To whom correspondence should be addressed

Table of Contents

Figure S1: Schematic representation of the proposed proteogenomics pipeline.
Figure S2: Prediction errors for confident target peptide-to-spectrum matches.
Figure S3: Comparison of predicted retention time and RT apex for decoy and confident target hits.
Figure S4: Q-Q plot of search engine score distributions between extended and canonical databases.
Figure S5: Q-Q plots of PSM features distributions between Extended DB and UniProt DB.
Figure S6: Violin plots of PSM features distributions between extended and canonical databases.

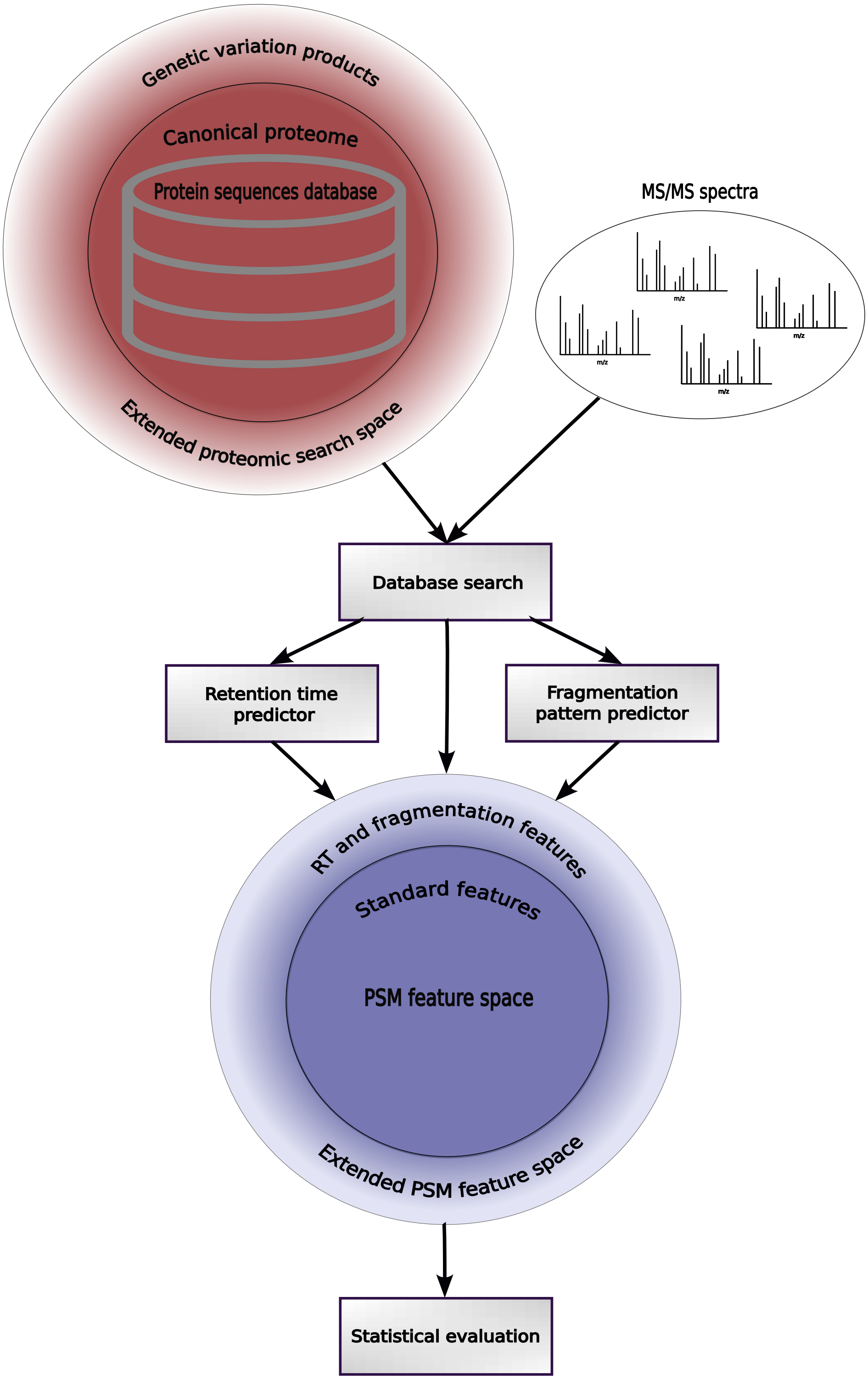

Figure S1: Proposed proteogenomics pipeline.

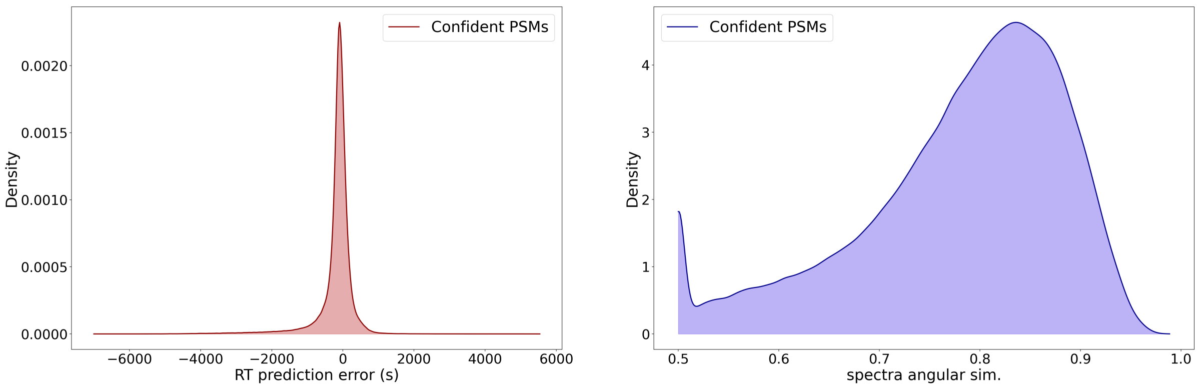

(A) **(B)**

Figure S2: Density plots of peptide feature prediction errors obtained from the search against the extended database for confident target hits. The two figures represent the agreement between measured and predicted features with peptide (A) retention time and (B) fragmentation obtained using DeepLC and MS^{2}PIP, respectively. The distributions plotted here consider the target PSMs accepted as confident by Percolator using the standard set of features. See methods for details on how these features are computed.

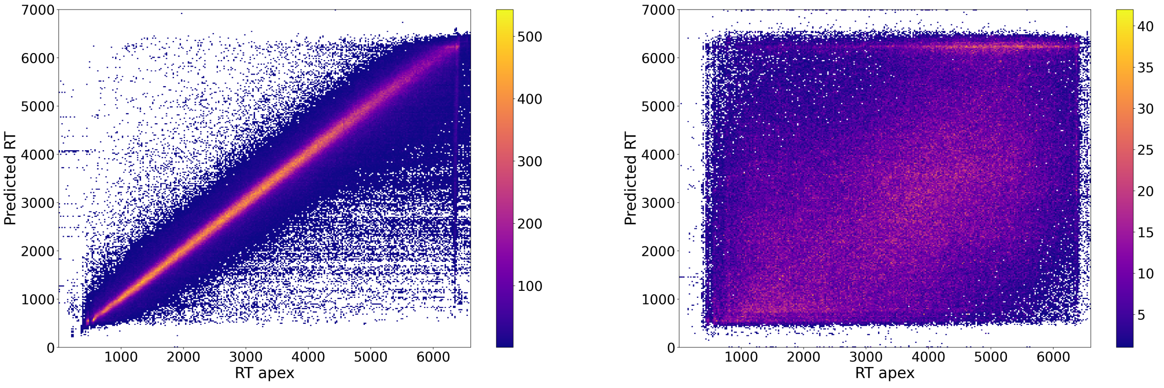

(A) **(B)**

Figure S3: Heatmaps of predicted retention time vs. RT apex obtained from the search against the extended database. The two figures represent the agreement between the RT apex and predicted retention time for (A) confident target hits and (B) decoy hits using DeepLC. The target hits plotted in (A) consider the PSMs accepted as confident by Percolator using the standard set of features. For the few PSMs where the predictions were either negative or larger than 7,000 seconds, the outlier values of 0 and 7,000 were plotted instead.

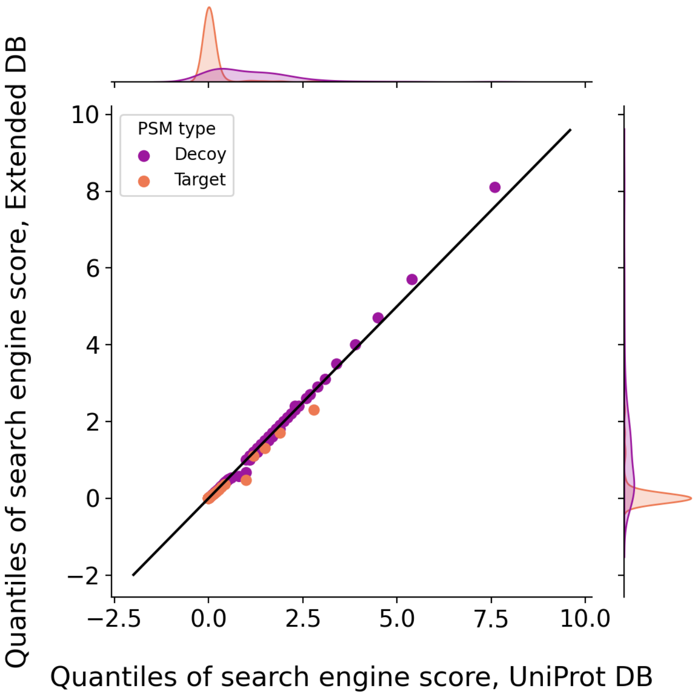

Figure S4: Comparison of search engine score distributions between extended and canonical databases. Q-Q plots that compare the distributions of the search engine score of target and decoy PSMs at a 5% FDR from the variant-aware Ensembl database and UniProt DB.

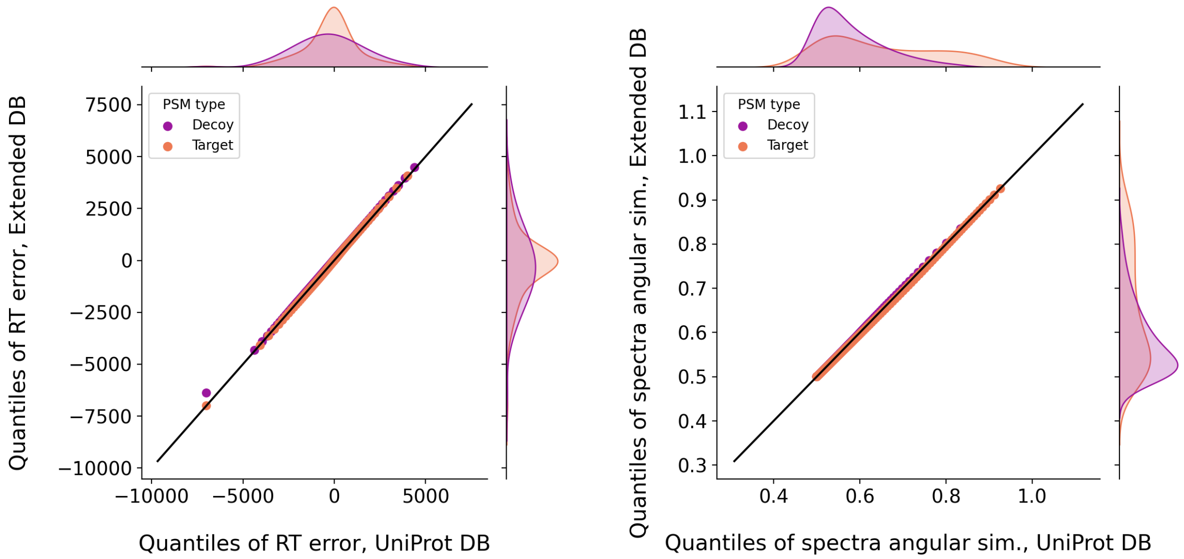

(A) **(B)**

Figure S5: Comparison of PSM features distributions between extended and canonical databases. Q-Q plots that compare the distributions of the retention time error (A) and spectra angular similarity (B) between measured and predicted values of identified target and decoy peptide sequences from the Extended DB and UniProt DB.

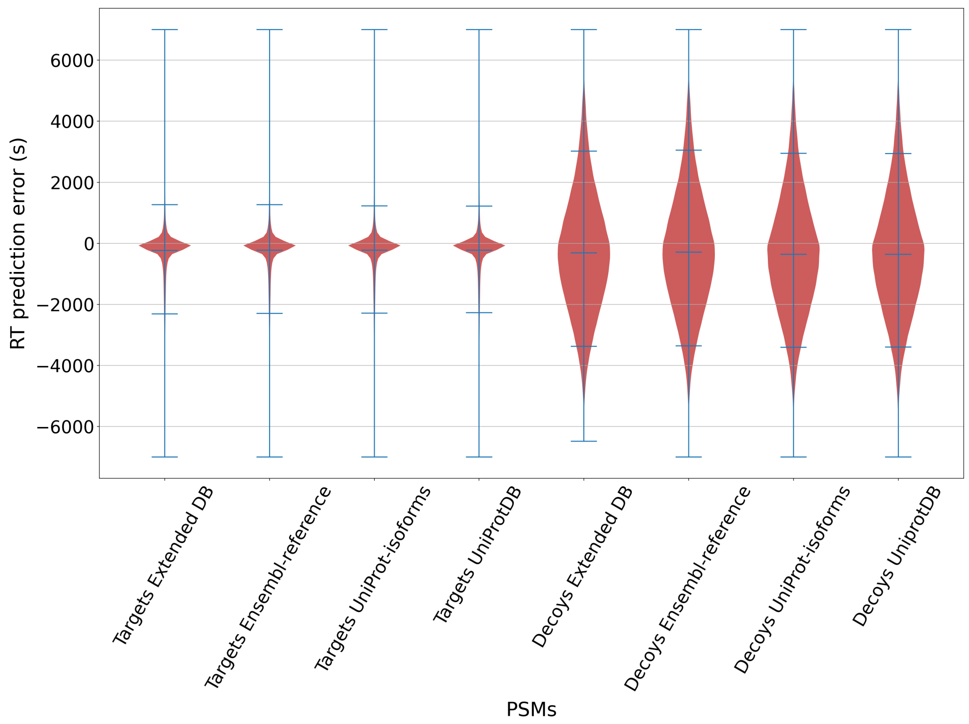

(A)

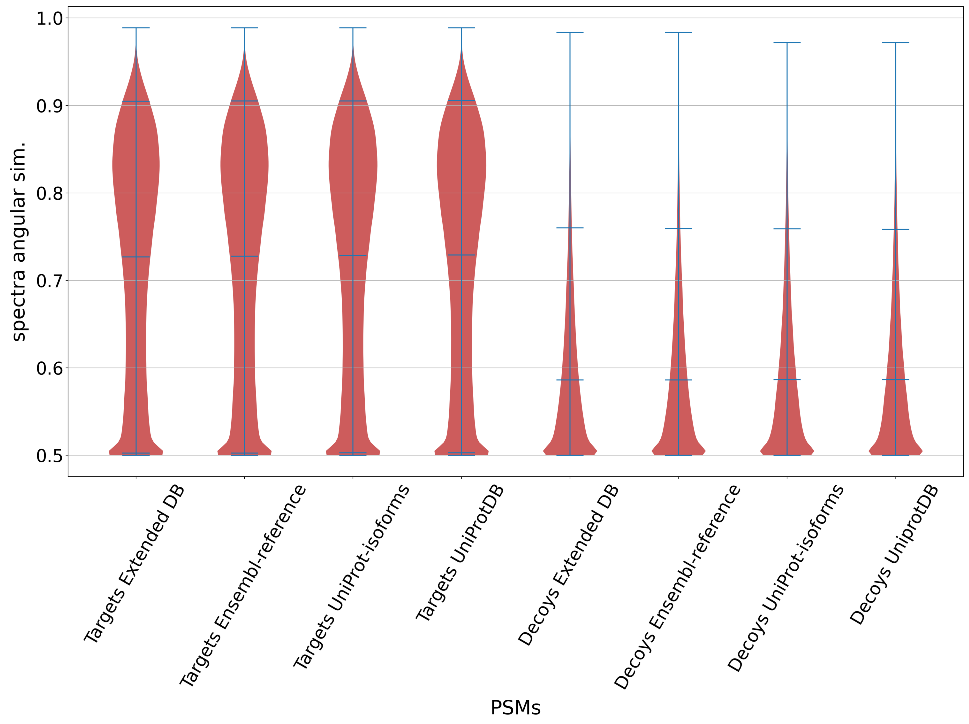

(B)

Figure S6: Violin plots of PSM features obtained from the search against the four protein databases for target and decoy hits. The two features presented in these figures represent the agreement of the matches with peptide (A) retention time and (B) fragmentation obtained using DeepLC and MS^2PIP, respectively. See methods for details on how these features are computed.
